# Participatory species naming as a tool for public engagement and conservation

**DOI:** 10.1093/biosci/biag040

**Published:** 2026-04-09

**Authors:** Cene Fišer, Maja Zagmajster

**Affiliations:** Department of Biology, Biotechnical Faculty, University of Ljubljana, Ljubljana 1000, Slovenia; Department of Biology, Biotechnical Faculty, University of Ljubljana, Ljubljana 1000, Slovenia

Recent discussion on the use of eponyms (Guedes et al. 2023, Jost et al. 2023, Thiele 2023) highlights that species naming can be a powerful tool for public engagement and biodiversity awareness. Less often acknowledged is that creativity in species naming can also support fundraising, attract public attention, and promote gender equity (Heard and Mlynarek 2023, Pétillon et al. 2025). Linnaean binomials provide convenient identifiers for species hypotheses (Thiele and Yeates 2002, Hey 2006), linking taxonomic expertise with broader biodiversity research. If etymology can bridge biodiversity research with society at large, taxonomy has the potential to become an integral part of science communication, particularly in raising awareness about the importance of local biota. Although debates on eponyms—species names derived from personal names—have highlighted problematic practices (Guedes et al. 2023, Jost et al. 2023, Thiele 2023), other examples of species naming that actively involve the public have received little attention.

Naming a species is the final step in the formal description process and lies largely at the discretion of taxonomists (Heard and Mlynarek 2023). Typically, researchers select the species name, and information about the discovery is disseminated through various media or organized initiatives such as the annual “WoRMS Top-Ten Marine Species” (WoRMS 2025). A few cases deviate from this pattern. During exploration of Europe’s largest cave system, the Hölloch Cave System in Switzerland, cavers discovered three previously unknown amphipod species. To acknowledge these discoveries, members of the cave society were invited to propose species names for the new taxa (Fišer et al. 2017). Similarly, in Bosnia and Herzegovina, a conservation-oriented project uncovered a new cave shrimp species. At the project’s closing symposium in Trebinje, the local community voted for one of three proposed names, either by anonymous cards or through a Facebook poll (Jugovic et al. 2026). Another shrimp species was named through a local radio initiative in Berlin, which invited listeners to suggest epithets, resulting in 97 proposals that informed the final choice (Klotz et al. 2023). Exclusively online engagement determined the name of a deep-sea chiton, in which the Senckenberg Ocean Species Alliance collaborated with a YouTuber to invite global participants to propose and justify epithets through social media, involving 8000 participants (SOSA et al. 2026). These cases illustrate how participatory taxonomy can engage non-biologists and diverse social groups, fostering public interest in species discoveries and awareness of local biodiversity.

We are living through a mass extinction, much of it driven by human activity. Every component of biodiversity science should contribute to raising public awareness and improving stewardship of the remaining biodiversity. Taxonomy, often perceived as a purely technical discipline, has advanced conceptually, analytically, and technically into an interdisciplinary, hypothesis-driven field reliant on highly trained experts (Zamani et al. 2022). Yet it also offers opportunities for citizen science at both the earliest and latest stages of research—specimen collection (Alther et al. 2021) and species naming (Fišer et al. 2017, Jugovic et al. 2026). By integrating social and communication sciences, taxonomy can become a powerful tool for raising awareness, guiding conservation, and informing policies that protect our remaining biodiversity.
